# Gastric and esophageal cancer in China 2000 to 2030: Recent trends and short‐term predictions of the future burden

**DOI:** 10.1002/cam4.4586

**Published:** 2022-02-11

**Authors:** Jiachen Zhou, Rongshou Zheng, Siwei Zhang, Ru Chen, Shaoming Wang, Kexin Sun, Minjuan Li, Shaoyuan Lei, Guihua Zhuang, Wenqiang Wei

**Affiliations:** ^1^ Department of Epidemiology and Biostatistics School of Public Health, Xi'an Jiaotong University Health Science Center Xi'an China; ^2^ National Central Cancer Registry, National Cancer Center/National Clinical Research Center for Cancer/Cancer Hospital Chinese Academy of Medical Sciences and Peking Union Medical College Beijing China

**Keywords:** China, esophageal cancer, gastric cancer, prediction, temporal trends

## Abstract

**Background:**

Gastric and esophageal cancer (GEC) have made a great contribution to the cancer burden in China. This study aims to report GEC incidence and mortality trends in 2000–2015 and their predictions to 2030.

**Methods:**

We collected GEC data from 22 cancer registries for Joinpoint temporal trend analysis between 2000 and 2015 and average annual percent change was calculated. Based on the historical changes, combined with the 2015 GEC national incidence and mortality rate, the rate from 2016 to 2030 was predicted grouped by sex and age. The crude rate, standardized rate, and cumulative rate were calculated. The number of cases were obtained by multiplying the United Nations' World Population Prospects and the predicted rate of corresponding years. Attribution changes between 2015 and 2030 were apportioned into demographics and risk factors.

**Results:**

There were decreasing trends of age‐standardized incidence rate world (ASIRW) and age‐standardized mortality rate world (ASMRW) during 2000–2015 in China (*p* < 0.05), the decline was more significant for the age group of 40–49 years in men and the age group of 50–59 years in women. It was predicted that in 2030, about 549,724 new cases and 394,576 deaths of GEC would occur in China. Compared with 2015, the numbers of new GEC cases and deaths in 2030 decreased by 15.24% and 17.62%, respectively. From 2020 to 2030, GEC ASIRW and ASMRW were predicted to decline from 24.98 to 17.47 and from 17.41 to 11.82 per 100,000, respectively. The number of new cases decreased by about 15.24% with changing demographic (44.48%) and risk (−59.72%) and the number of deaths decreased by about 17.62% with changing demographic (47.18%) and risk (−64.80%).

**Conclusions:**

Although GEC incidence and mortality rates showed downward trends, the disease burden remains heavy in China. The current prevention and control strategy are effective which need to be carried on.

## Graphical abstract

We systematically analyzed the long‐term trends of GEC incidence and mortality by age and gender and found that the rates in most age groups showed a downward trend. After attribution analysis, it was found that demographic factors, including age structure and population size, led to an increase of 44.48% in the number of new cases and 47.18% in deaths. The results indicated that the main reason for the future significant impact on the GEC burden is the aging population.

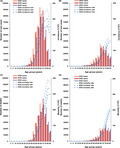

## 1 INTRODUCTION

Gastric and esophageal cancers (GEC) are global public health challenges, with estimates of 1.69 million new cases and 1.31 million deaths in 2020 worldwide.^1^ Approximately 47.42% of all GEC occur in China.^2^ Although the survival of GEC has been improved in recent years, the 5‐year survival rate is still maintained at about 30% in China.^3^

To improve the early diagnosis rate and early treatment rate, a number of population‐based screening projects were implemented in high‐risk areas of China, including Cancer Screening Program in Rural since 2005 and Cancer Screening Program in Urban since 2012. In 2019, the Cancer Screening Program in Rural has covered 196 counties, and has screened more than 1.5 million high‐risk people in high‐risk areas of GEC. Long‐term follow‐up studies in China found that as long as one‐third of the residents in high‐risk areas were screened, the incidence and mortality of esophageal cancer can be effectively reduced, indicating that endoscopic screening is an effective method for intervention and control of GEC.^4^ Although population screening has made significant progress, relevant researches are still limited to high‐risk areas. There is still a lack of evidence on the effectiveness of GEC screening programs for non‐high‐risk population. And the changes in the overall burden of GEC in China are still unclear.

Previous study used data extracted from the Cancer Incidence in Five Continents series to explore long‐term trends of gastric and esophageal cancer in 20 world regions.^5^ Shao also reported a trend analysis of gastric cancer, esophageal cancer, and colorectal cancer from 1991 to 2015 in Yangzhong.^6^ The National Central Cancer Registry (NCCR) of National Cancer Center has estimated the cancer burden in 2015, based on data from 368 registries across the country.^7^ However, China still lacks long‐term trend analysis and burden forecasting for GEC in China.

Predicting the future disease burden of GEC has a pivotal role in cancer control policy.^8^ Thus, in this study, we aim to present the recent GEC incidence and mortality trends and short‐term predictions to the year 2030 in China. These findings can provide evidence for GEC cancer prevention and control policymaking over the next decades.^9^

## 2 MATERIALS AND METHODS

### 2.1 Data Sources and quality control

According to *Measures for the Administration of Cancer Registration*, the NCCR collects cancer registration data from 31 provinces, autonomous regions and municipalities in China.^10^ All hospitals, medical and health institutions in administrative regions are required to submit cancer records to local cancer registries. All cancer registries are classified where prefecture‐level cities and above are divided into urban areas, and county‐level cities and below are divided into rural areas. As of August 2018, a total of 501 cancer registries submitted cancer registry data of 2015.^7^ NCCR collected, evaluated and selected 368 registries' data in the pool data according to the *Chinese Guideline for Cancer Registration (2016)* and International Agency for Research on Cancer (IARC)/International Association of Cancer Registries relevant evaluation criteria.^11^, ^12^, ^13^ Quality control included the validity, reliability, completeness, and comparability of all cancer registry data. A series of indexes were taken into consideration, including the mortality to incidence (M/I) ratio, the percentage of cases morphologically verified (MV), the percentage of death certificate‐only cases (DCO), the percentage of the diagnosis of unknown basis (UB), and the stability of cancer trends over years.^7^ According to International Statistical Classification of Diseases and Related Health Problems 10th Revision (ICD‐10), gastric cancer and esophageal cancer cases coded as C15 and C16 were extracted for analysis. NCCR estimated nationwide number of cases, crude rates, and age‐standardized rates by age and gender.

Twenty‐two cancer registries set up before the 1990s in China can provide qualified registry data, including 11 urban and 11 rural registries. Population‐based registration data from these registries were used for long‐term cancer burden monitoring. We extracted data from these 22 registries from 2000 to 2015 for trend analysis. NCCR performed quality control on the data in the same way, and data met the quality requirements were selected in the data pool. According to ICD‐10, gastric cancer and esophageal cancer cases coded as C15 and C16 were extracted for analysis. Cancer coding methods and principles were consistent each year. NCCR calculated age‐standardized incidence rate world (ASIRW) and age‐standardized mortality rate world (ASMRW) by gender and age every year.

### 2.2 Statistical analysis

For temporal trend analysis, cancer birth cohort and Joinpoint model were conducted using the data from 22 cancer registries, 2000 to 2015. The rate was fitted by the formula in Joinpoint model,

$$\ln\left(y\right)=\alpha +\beta x+\varepsilon$$

where y is the incidence/mortality rate, α is the constant term, β is the regression coefficient, and ε is the random error term. Trends were expressed as annual percentage change (APC) and average annual percentage change (AAPC) by gender and age groups.^14^, ^15^, ^16^

$$\mathrm{APC}=\left(e^{\beta }-1\right)\times 100\%$$

$$\text{AAPC}=\left\{\;\exp\sum w_{i}b_{i}/\sum w_{i}-1\right\}\times 100\%$$

Among them, $b_{i}$ represented the slope coefficient of each trend, $w_{i}$ represented the duration of each trend, and the Z test was used to analyze statistical differences through the JoinPoint 4.3.1.0 software.^14^

To reduce the possibility of reporting spurious changes in trends over the period, models were restricted to a maximum of two joinpoints.^17^

We selected the trend analysis result of the last segment as the future trend change rateβ, year as x and ε as the random error term. Use y=α+βx+ε and the incidence and mortality of GEC in 2015 to predict the rate in 2016–2030, grouped by age (0 to 80 by 5 years, 85+) and gender (male/female). The cumulative incidence/mortality rate (0–74 years old) was calculated.^11^ The number of new cases were predicted by multiplying the predicted incidence rates by the population forecasts of China from the *World Population Prospects 2019* made by the United Nation. The calculation of the number of deaths was consistent with the count algorithm of the number of new cases.

The change of cases was influenced by risk of GEC and population age structure and size. Let $N_{\mathrm{ras}}\;$be the number of cases given a risk *r*, an age structure *a* and a population size *s*, where *r*, *a,* and *s* were leveled for the observed 2015 (o) or the predicted 2030 (f).^18^ $N_{\mathrm{ooo}}$ applied the rates, age structure, and population size of 2015, and $N_{\mathrm{fff}}$ was the predicted number of cases in 2030. $N_{\mathrm{off}}\;$applied the rates of 2015 and age structure and population size of 2030. $N_{\mathrm{oof}}\;$applied the rates and age structure of 2015 and population size of 2030. Thus, the change between 2015 and 2030 was decomposed into three components.

$$\Delta_{\text{total}}=N_{\mathrm{fff}}-N_{\mathrm{ooo}}$$

$$\Delta_{\text{total}}=\left(N_{\mathrm{fff}}-N_{\mathrm{off}}\right)+\left(N_{\mathrm{off}}-N_{\mathrm{ooo}}\right)$$

$$\Delta_{\text{total}}=\left(N_{\mathrm{fff}}-N_{\mathrm{off}}\right)+\left(N_{\mathrm{off}}-N_{\mathrm{oof}}\right)+\left(N_{\mathrm{oof}}-N_{\mathrm{ooo}}\right)$$

$$\Delta_{\text{total}}=\Delta_{\text{risk}}+\Delta_{\mathrm{age}}+\Delta_{\text{size}}$$

The total change percentage was $\left(\frac{\Delta_{\text{total}}}{N_{\mathrm{ooo}}}\right)\times 100$. Change of risk, age structure, and population size were calculated in the same way. The Chinese population in 2000 and Segi's world population were used to calculate the age‐standardized rate China and world.^19^ We applied SAS software (Version 9.4, SAS Institute Inc.) for statistical analysis.

## 3 RESULTS

### 3.1 Temporal trends between 2000 and 2015

**Figure 1:**
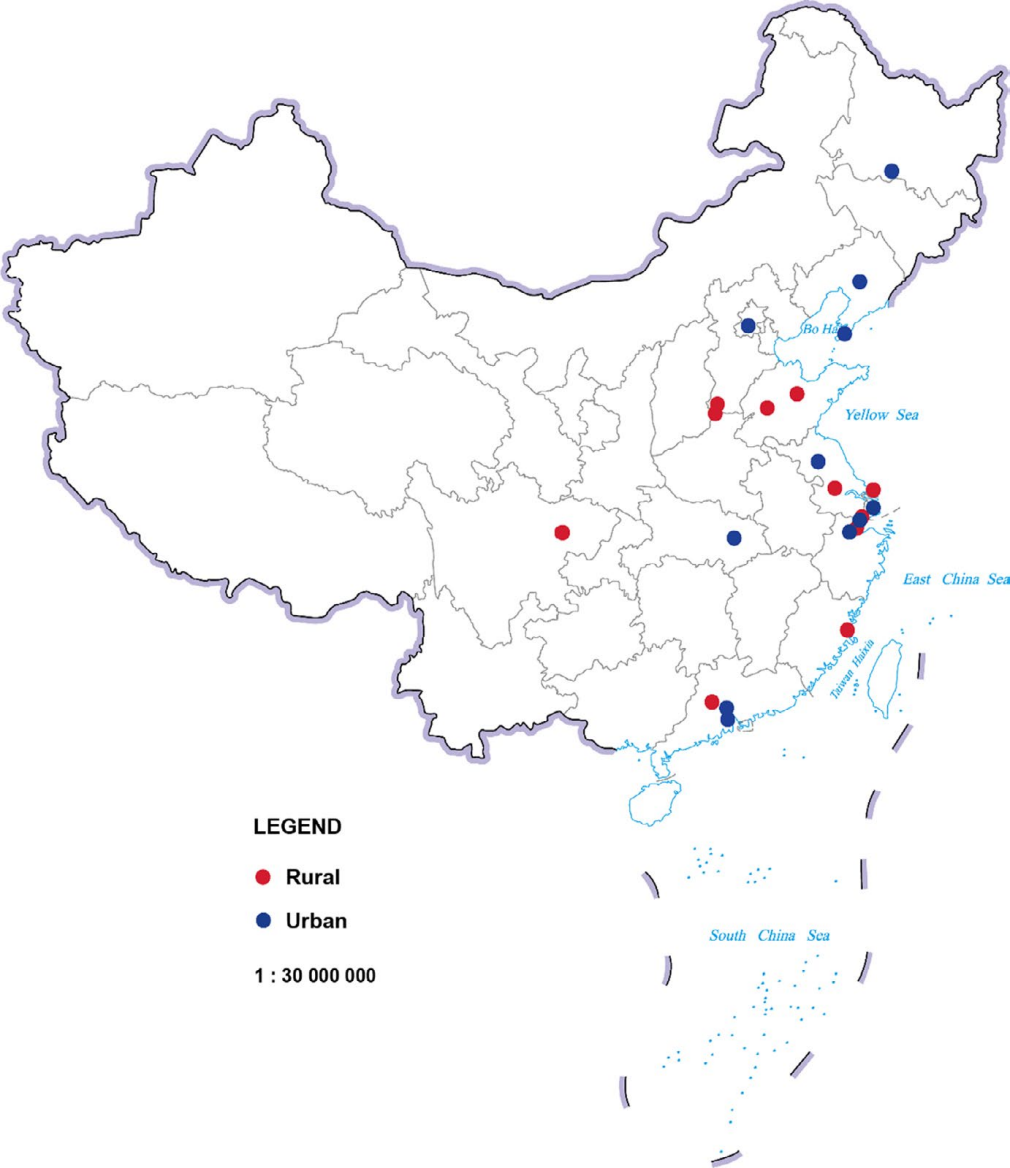
Geographical distribution of the 22 cancer registries in China

About 46.59 million people in the Chinese main population area were covered by 22 cancer registries, accounting for 3.39% of the national population at the end of year 2015 (Figure 1). Between 2000 and 2015, the ASIRW of GEC decreased by an average of 3.5% (−4.4% to −2.7%) (Z = −7.9, *p* < 0.05) per year. Male incidence AAPC was −3.4% (−3.9% to −2.9%) (Z = −14.3, *p* < 0.05) and female AAPC was −3.9% (−4.2% to −3.6%) (Z = −25.3, *p* < 0.05). The average decline in ASMRW was 4.2% (−4.7% to −3.7%) (Z = −16.4, *p* < 0.05). AAPCs in male and female were − 4.0% (−4.5% to −3.5%) (Z = −14.8, *p* < 0.05) and − 5.1% (−5.9% to −4.2%) (Z = −11.3, *p* < 0.05), respectively (Table 1). The declining trends of ASIRW were similar for male and female, while the declining trends of ASMRW were more obvious for females than for males. The GEC incidence of male showed a significant downward trend during 2000–2003, the downward trend slowed down during 2003–2010, and a significant downward trend appeared again during 2010–2015, but the rate of decline was slower than the first period of time. The female incidence rate showed a persistent downward trend, instead. The GEC mortality rate of male and female showed a rapid‐slow‐rapid decline process as that of male incidence. Analysis of age‐specific incidence and mortality rates showed that the decrease was observed in all age groups, but the age group 40–49 years in male and the age group 50–59 years in female decreased more significantly (Table S1 and S2). Urban incidence AAPC was −3.6% (−4.2% to −3.0%) (Z = −11.7, *p* < 0.05) and rural AAPC was −2.1% (−2.7% to −1.5%) (Z = −6.9, *p* < 0.05). Mortality AAPCs in urban and rural were − 4.2% (−4.6% to −3.7%) (Z = −18.0, *p* < 0.05) and − 2.6% (−3.3% to −2.0%) (Z = −8.1, *p* < 0.05), respectively. The decline in urban areas was more significant than that in rural areas. The rate of urban showed a rapid‐slow‐rapid decline process while the rural rate showed a more obvious downward trend in the second period.

**Table 1:** JoinPoint analysis of the trends of the ASIRW and ASMRW of GEC in 22 cancer registration areas of China, 2000 to 2015

	Trend 1		Trend 2		Trend 3		2000–2015
	Period	APC (95% CI) (%)	Period	APC (95% CI) (%)	Period	APC (95% CI) (%)	AAPC (95% CI) (%)
ASIRW							
Both	2000–2004	−4.9^*^(−7.0 to −2.8)	2004–2009	−1.7(−3.9 to 0.5)	2009–2015	−4.1^*^(−5.2 to −3.0)	−3.5^*^(−4.4 to −2.7)
Male	2000–2003	−5.7^*^(−7.4 to −3.9)	2003–2010	−1.9^*^(−2.5 to −1.3)	2010–2015	−4.1^*^(−4.9 to −3.3)	−3.4^*^(−3.9 to −2.9)
Female	2000–2015	−3.9^*^(−4.2 to −3.6)	—	—	—	—	−3.9^*^(−4.2 to −3.6)
Urban	2000–2003	−6.2^*^ (−8.4 to −4.0)	2003–2009	−1.6^*^(−2.6 to −0.5)	2009–2015	−4.2^*^(−5.0 to −3.4)	−3.6^*^(−4.2 to −3.0)
Rural	2000–2007	0.3(−0.7 to 1.4)	2007–2015	−4.1^*^(−5.0 to −3.3)	—	—	−2.1^*^(−2.7 to −1.5)
ASMRW							
Both	2000–2003	−7.3^*^(−9.2 to −5.4)	2003–2010	−2.7^*^(−3.4 to −2.0)	2010–2015	−4.5^*^(−5.4 to −3.6)	−4.2^*^(−4.7 to −3.7)
Male	2000–2003	−7.3^*^(−9.2 to −5.3)	2003–2011	−2.5^*^(−3.0 to −1.9)	2011–2015	−4.5^*^(−5.8 to −3.2)	−4.0^*^(−4.5 to −3.5)
Female	2000–2004	−6.9^*^(−9.0 to −4.7)	2004–2010	−3.4^*^(−5.0 to −1.8)	2010–2015	−5.6^*^(−7.1 to −4.0)	−5.1^*^(−5.9 to −4.2)
Urban	2000–2003	−8.0^*^(−9.7 to −6.3)	2003–2009	−1.8^*^(−2.6 to −1.0)	2009–2015	−4.6^*^(−5.2 to −4.0)	−4.2^*^(−4.6 to −3.7)
Rural	2000–2006	−0.6(−2.0 to 0.8)	2006–2015	−4.0^*^(−4.7 to −3.2)	—	—	−2.6^*^(−3.3 to −2.0)

Abbreviations: 95% CI, 95% confidence interval; AAPC, average annual percent change; APC, annual percent change; ASIRW, age‐standardized incidence rate world; ASMRW, age‐standardized mortality rate world; GEC, gastric and esophageal cancer.

^*^ The APC and AAPC are significantly different from zero (*p* < 0.05).

**Figure 2:**
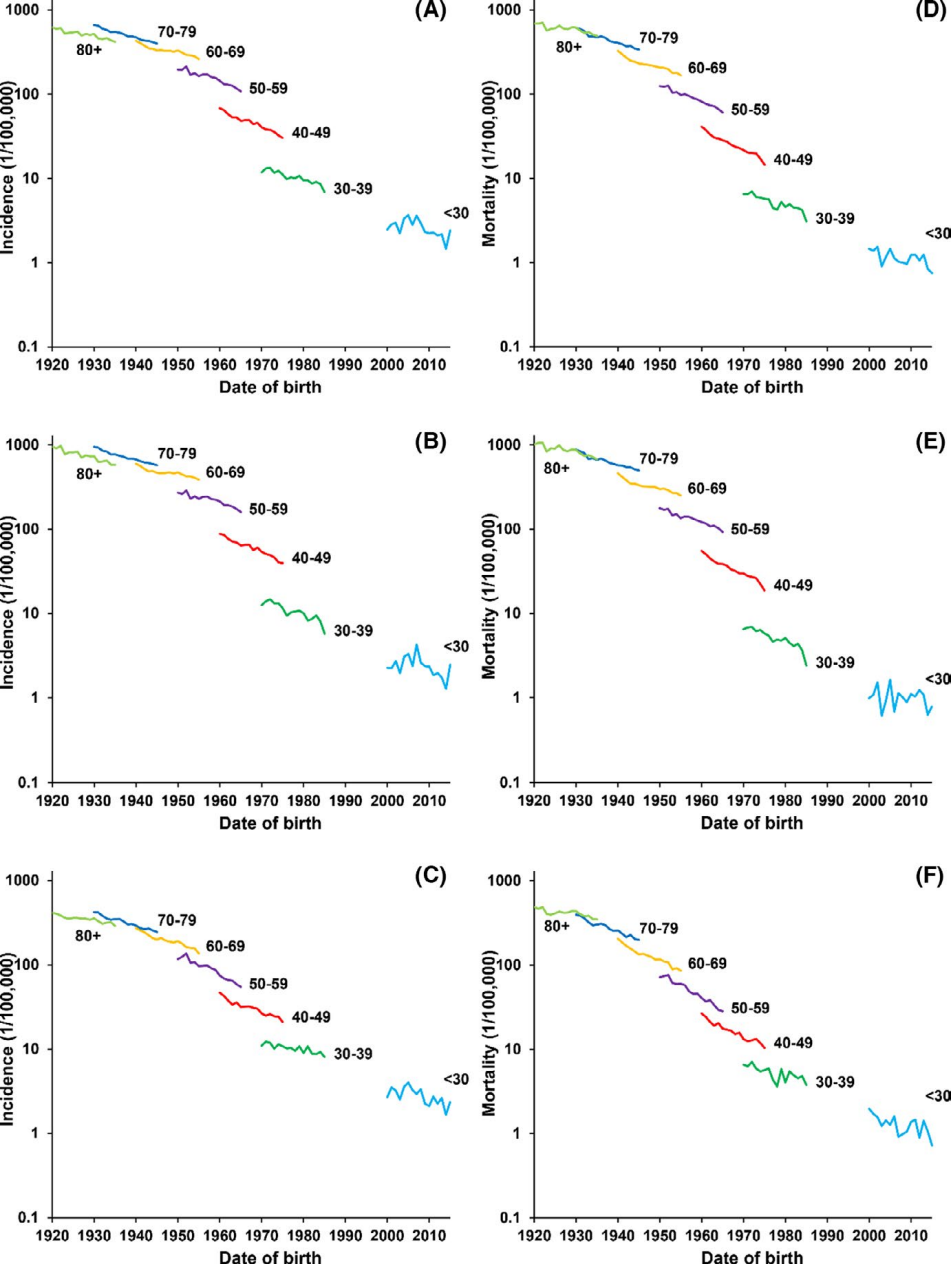
GEC rates of different birth cohorts by age groups in China. GEC, Gastric and esophageal cancer. (A) Both incidence; (B) Male incidence; (C) Female incidence; (D) Both mortality; (E) Male mortality; (F) Female mortality

The incidence rate between 39 and 79 years old increased significantly with age in both gender (Figure 2). However, these age groups showed significant decreasing trends between 2000 and 2015. The age groups of 40–49 and 50–59 years had the most obvious downward trends. Compared with the incidence rates, mortality rates decreased more significantly in each age group. Similarly, the age groups of 40–49 and 50–59 years had significant downward trends than other age groups, both male and female.

### 3.2 Predictions in 2020 and 2030

In 2020 and 2030, a total of 573,165 and 549,724 new cases of GEC have been estimated. The national crude incidence rates are 39.82/100,000 and 37.54/100,000, respectively. The age‐standardized incidence rate China (ASIRC) and ASIRW are 24.97/100,000 and 24.98/100,000 in 2020, and 17.49/100,000 and 17.47/100,000 in 2030 (Table 2).

**Table 2:** Incidence and mortality of GEC in 2020 and 2030

Year	Rate	Sex	Cases (×10^4^)	Crude rate (1/100000)	ASRC (1/100000)	ASRW (1/100000)	Cumulative rate 0–74 (%)
2020	Incidence	Both	57.32	39.82	24.97	24.98	3.10
		Male	40.72	55.15	36.05	36.31	4.65
		Female	16.60	23.68	13.89	13.66	1.60
	Mortality	Both	40.40	28.07	17.48	17.41	2.00
		Male	28.68	38.85	25.54	25.59	3.07
		Female	11.72	16.72	9.42	9.23	0.96
2030	Incidence	Both	54.97	37.54	17.49	17.47	2.20
		Male	39.13	52.30	25.51	25.69	3.39
		Female	15.84	22.12	9.47	9.25	1.05
	Mortality	Both	39.45	26.95	11.84	11.82	1.32
		Male	28.03	37.47	17.54	17.62	2.12
		Female	11.42	15.95	6.13	6.01	0.56

Abbreviations: ASRC, age‐standardized rate China; ASRW, age‐standardized rate world; GEC, gastric and esophageal cancer.

In 2020 and 2030, 404,011 and 394,577 deaths of GEC have been estimated. The crude mortality rates are 28.07/100,000 and 26.95/100,000, respectively. The age‐standardized mortality rate China (ASMRC) and ASMRW are 17.48/100,000 and 17.41/100,000 in 2020 and 11.84/100,000 and 11.82/100,000 in 2030.

In 2020, the age group of 65–69 years has the largest number of incidence cases in males, while in 2025 and 2030 it is the age group of 70–74 and 65–69 years. The number of cases in the age group 75–79 years will increase with the time and reach the highest in 2030. However, the number of cases in the age group of 50–54 years will decrease with the time and reach the lowest in 2030. In females, the age group of 65–69 years has the largest number of cases in 2020, while in 2030 it will be the age group of 65–69 and 75–79 years.

**Figure 3:**
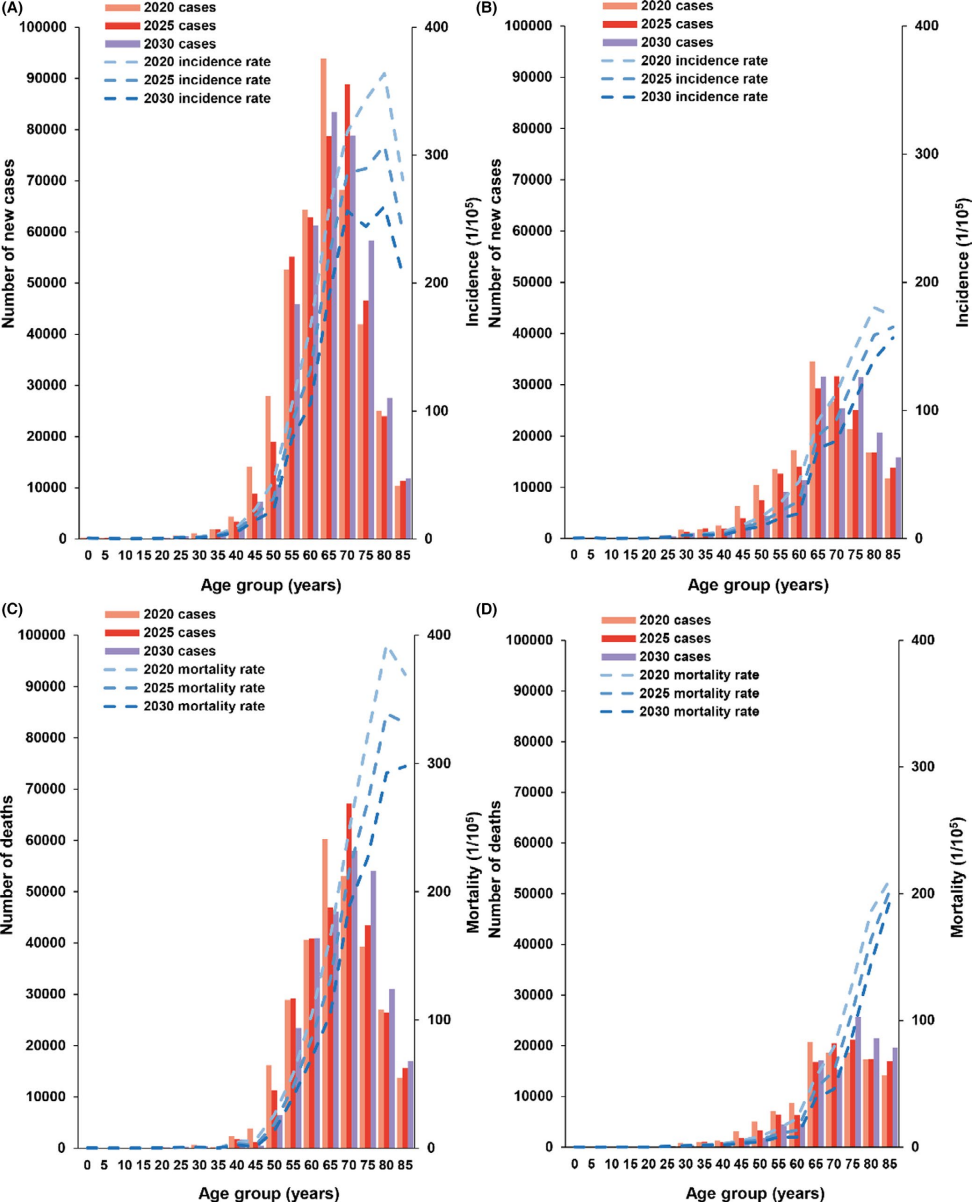
Age‐specific incidence and mortality rates and cases of GEC in China, 2020, 2025, and 2030. GEC, Gastric and esophageal cancer. (A) Male incidence; (B) Female incidence; (C) Male mortality; (D) Female mortality

In males, the age group of 65–69 years has the largest number of deaths in 2020, while in 2025 and 2030 the age group of 70–74 years will have the largest number. In males, the number of deaths in the age group of 75–79 years will increase with time and reach the highest in 2030. The lag of mortality peak is also observed in females. The maximum value of number of deaths will occur in the age group of 75–79 years in 2025 and 2030 (Figure 3).

### 3.3 Change between 2015 and 2030

Compared with 2015 and 2020, GEC ASIRW and ASMRW will further decrease in both gender in 2030. ASIRW will drop to 17.47/100,000, a 41.47% decrease compared to 2015 (29.85/100,000). The number of new GEC cases will decrease by about 15.24% with changing incidence rates among which changing demographic and risk contribute 44.48% and −59.72%, respectively. ASMRW will drop to 11.82/100,000, a 44.43% decrease compared to 2015 (21.27/100,000). The number of GEC deaths will decrease by about 17.62% with changing mortality rates among which changing demographic and risk contribute 47.18% and −64.80%, respectively.

In 2030, the ASIRW and ASMRW in males will drop to 25.69/100,000 and 17.62/100,000, and the ASIRW and ASMRW in females will drop to 9.25/100,000 and 6.01/100,000. The overall change in female attribution is greater than that of males, including an increase in demographic and a decrease in risk. Although the number of deaths among males are still twice that of females, the change in attribution is smaller than that of females (Table 3).

**Table 3:** Predicted number of new GEC cases and deaths in China and changes between 2015 and 2030 apportioned into changes because of risk and demographics by gender

Sex	Number of new cases or deaths				Changes between 2015 and 2030 (%)				
	2015	2020	2025	2030	Total Change	Change due to risk	Change due to demographic	Change due to age structure	Change due to population size
Incidence									
Both	648,600	573,165	563,758	549,724	−15.24	−59.72	44.48	37.95	6.53
Male	458,200	407,178	402,647	391,311	−14.60	−56.73	42.14	35.88	6.26
Female	190,400	165,987	161,111	158,413	−16.80	−63.03	46.23	39.42	6.80
Mortality									
Both	479,000	404,011	399,001	394,577	−17.62	−64.80	47.18	40.66	6.52
Male	338,300	286,802	285,059	280,342	−17.13	−61.38	44.25	38.01	6.24
Female	140,700	117,209	113,942	114,235	−18.81	−68.05	49.24	42.41	6.82

Abbreviation: GEC, gastric and esophageal cancer.

## 4 DISCUSSIONS

Our study provides evidence for China GEC trend analysis and forecasting burden in the next 15 years. We systematically analyzed the long‐term trends of GEC incidence and mortality by age and gender and found that the rates in most age groups showed a downward trend. Compared with 2015, the number of new cases and deaths will decrease in the next decades in both sexes. However, after attribution analysis, it was found that demographic factors, including age structure and population size, led to an increase of 44.48% in the number of new cases and 47.18% in deaths. The results indicated that the main reason for the future significant impact on the GEC burden is the aging population.

The declining trends of different ages were different. The results showed that the declining trends of the younger age group was more obvious than the older age group. After age‐specific rate trend analysis, we found that the change of the age group of 40–59 years was generally greater than that of over 60 groups. The decline was most obvious in men and women in the age group of 40–49 and 50–59 years, respectively. Although trend analysis of standardized rates showed downward trends of GEC, the crude incidence and mortality rates remained stable. Attribution analysis revealed that the incidence will totally decrease by 15.24%, but the age structure and population size will increase cases by 37.95% and 6.53%, respectively. The comparison of the number of new cases by age in 2020, 2025, and 2030 showed that the peak of new cases gradually shifts to older age groups, while the number of cases in the lower age group is decreasing year by year, which was likely to lead to an increase in the average age of onset.

Melina uses data from 42 registries in 12 developed countries of Cancer Incidence in Five Continents to predict the esophageal squamous cell carcinoma (ESCC) trend by age‐period‐cohort models which is predicted to continue decreasing in most countries up to 2030.^20^ However, the number of new esophageal adenocarcinoma cases is expected to increase rapidly from 2005 to 2030 in all studied countries. China's esophageal cancer is still dominated by ESCC, which accounts for 86.3%. In this way, we are consistent with the international forecast. Joliat^21^ also predicts Switzerland GEC trend until 2029. The incidence and mortality of esophageal cancer rose slightly but the rates of gastric cancer both decreased significantly. After the European population standardization, the overall GEC also shows a downward trend. However, the downward trend in the Swiss rate is longer and larger.

Both age‐period‐cohort models and Joinpoint model are used to fit the trend of the rate. The advantage of age‐period‐cohort models is that they can simultaneously adjust the three factors of age, period, and cohort to predict the trend of disease. It is mainly used in descriptive epidemiology to analyze the trend of chronic disease and infectious diseases incidence and mortality. The Joinpoint model divides a long‐term trend line into several segments and describes each segment. It predicts the rate of each age group, and the short‐term results are more accurate. It does not have strict requirements on whether there is a trend in the data sequence itself, and is widely used in the trend analysis of cancer registration data.

Trends in this study were derived from 22 long‐term monitoring sites. They were all located in the south of the Heihe‐Tengchong Line (northeast‐southwest), covering the main population areas in China, and the trend change had certain reference value. GLOBOCAN 2020 predicted 802,930 GEC new cases and 674,924 deaths in China in 2020.^2^ In contrast, the number of cases in this study was estimated to be 28.62% and 40.14% lower than GLOBOCAN 2020. The difference between the two researches was that we used the data derived from 368 monitoring sites in 2015,^22^ while GLOBOCAN 2020 used the data derived from 90 monitoring sites between 2008 and 2012.^2^ China established the first batch of registries in high‐incidence areas to monitor key cancers. These registry data were reported to IARC via NCCR and were selected in Cancer Incidence in Five Continents at the same time. However, some registry data at these registries were higher than the whole country, and using these data tends to overestimate China's cancer burden. With the rapid increase in the coverage of cancer registration in China, the availability of effective monitoring data is also increasing. Facing the rapidly changing burden of cancer, the monitoring data extracted by the NCCR now is timely and can better reflect the cancer burden. By comparison, the data we predicted was closer to the real situation in China.

Comprehensively inferring from the results, population aging will be the main factor affecting the burden of GEC disease in the future. The continuous increase of the elderly population and the higher incidence of the elderly group will result in a large number of elderly GEC patients. Compared with the seventh census of China, the *World Population Prospects 2019* overestimate the total population size. Among them, the age group of 0–14 years and the age group of 15–59 years are both overestimated, while the age group of 60+ years is underestimated. Therefore, there are fewer adolescents than expected, and the actual trend of aging is faster and more obvious, which may lead to an underestimation of the number of the age group of 60+ years cases in our results.

The reason for the average age of onset change may be related to the decline in the incidence of the age group of 40–69 years and the increase in the elderly population. We also observed that age‐specific incidence and mortality fluctuates more in the age group of 40–69 years than in the other groups, partly because they are the target population for screening. This change from population will be the focus and difficulty of the control of GEC in the near future, which is both reflected in male and female. Although the incidence rate in 2020, 2025, and 2030 is decreasing year by year, the incidence change is limited. Thus, government intervention is still needed, especially for the senior age group.

GEC mainly occurs in backward rural areas and is related to smoking,^23^ alcohol drinking,^24^ and lack of nutrients.^25^ Trends in the overall burden of GEC over time are thought to be related to the promotion of urbanization, economic development, lifestyle changes, and screening programs. We found that the time of significant decline in rural areas was slower than that in urban areas, which is more likely to be related to differences in economic development. After quantifying the downward trends, most of the trends are divided into three segments which show a rapid‐slow‐rapid decline process. We speculate that it is because the changes in social development have the most obvious impact on the living environment and living habits at the beginning.

According to reports, the ASIRW's AAPC of esophageal cancer in male and female are −3.9% (−5.1% to −2.6%) (*p* < 0.05) and − 5.8% (−6.3% to −5.3%) (*p* < 0.05) from 2000 to 2015. Male ASIRW's AAPC of gastric cancer is −3.1% (−3.4% to −2.7%) (*p* < 0.05) and female is −2.8% (−3.1% to −2.6%) (*p* < 0.05).^7^ Except for female in gastric cancer, the downward trend of the two cancers is basically the same, of which esophageal cancer is more significant. Based on the characteristics of the two organs,^26^, ^27^, ^28^ we combined consideration of GEC trend burden changes to facilitate the actual work of cancer prevention and control. Endoscopy includes high‐risk area screening and clinical opportunistic screening is still the most suitable method for GEC prevention in China at this time, except for urbanization, economic development, and other nonpublic health measures. Only about one‐third to one‐half of the 22 cancer registries have started GEC screening, and the screening population is limited. In the overall data, there is no peak incidence which always shows up after screening. The cancer registration data is not detailed enough to determine whether the case comes from screening, follow‐up research is needed to further illustrate the relationship between screening and GEC trend.

The advantage of this study is that we used the latest 2015 cancer registration data as the baseline value, the estimates were closer to the real cancer burden in China, and the long‐term monitoring trends were reliable. The drawback was that we could not obtain official population estimates in China. However, we choose the United Nation's estimates of population for prediction, the short‐term deviations were limited and acceptable. In addition, no specific information about other risk factors has been collected in the attribution analysis, so currently only demographic and non‐demographic factors can be explained.

## 5 CONCLUSIONS

Although GEC has a downward trend in China, the incidence and mortality of GEC in China are at a high level compared with other countries or regions in the world. With the increasing aging of the population, the incidence of GEC will maintain a certain scale in the near future and the new cases gradually gather to the older age group. China still needs to strengthen the prevention and control of GEC, such as expanding the scope of endoscopic screening and the target age of the population in the grim situation.

## CONFLICT OF INTEREST

The authors have no conflict of interest.

## AUTHOR CONTRIBUTIONS

WW and GZ initiated, planned, and designed the study. JZ, RZ, and SZ had full access to all the data in the study. RC conducted the data acquisition, management, and analysis. KS, ML, and SL provided the statistical input for the data analysis. JZ, SW, and RZ drafted the manuscript. All authors interpreted the study results and critically revised the manuscript.

## ETHICS STATEMENT

As only anonymized data were obtained from the National Cancer Center for analyses, no patient consent process was involved.

## Supporting information


Table S1‐S2

## Data Availability

The datasets for this article are not publicly available because all data are under regulation of the National Cancer Center. Requests to access the datasets should be directed to Wenqiang Wei (weiwq@cicams.ac.cn).
