# Supplementary material for: Gastric and esophageal cancer in China 2000 to 2030: Recent trends and short‐term predictions of the future burden
Source: Cancer Med. 2022 Feb 11;11(8):1902–12. doi: 10.1002/cam4.4586 (PMC9041080; doi:10.1002/cam4.4586)
Supplement: Supplementary file 1 — Table S1‐S2 [file CAM4-11-1902-s001.docx]

**Supplementary:**

**Table S1** JoinPoint analysis of GEC age-specific incidence trends by gender, 2000 to 2015.

| Sex | Age  (year) | Trend 1 | |  | Trend 2 | |  | Trend 3 | |  | 2000-2015 |
| --- | --- | --- | --- | --- | --- | --- | --- | --- | --- | --- | --- |
|  |  | Period | APC  (95%CI) (%) |  | Period | APC  (95%CI) (%) |  | Period | APC  (95%CI) (%) |  | AAPC  (95%CI) (%) |
| Both | 0-39 | 2000-2015 | -3.2^a^(-3.9~-2.4) |  | — | — |  | — | — |  | -3.2 ^a^ (-3.9~-2.4) |
| Both | 40-49 | 2000-2015 | -4.7 ^a^ (-5.2~-4.3) |  | — | — |  | — | — |  | -4.7 ^a^ (-5.2~-4.3) |
| Both | 50-59 | 2000-2015 | -3.9 ^a^ (-4.5~-3.2) |  | — | — |  | — | — |  | -3.9 ^a^ (-4.5~-3.2) |
| Both | 60-69 | 2000-2004 | -5.9 ^a^ (-7.2~-4.5) |  | 2004-2010 | -0.7(-1.7~0.3) |  | 2010-2015 | -3.9 ^a^ (-4.9~-2.9) |  | -3.2 ^a^ (-3.7~-2.6) |
| Both | 70-79 | 2000-2015 | -3.2 ^a^ (-3.5~-3.0) |  | — | — |  | — | — |  | -3.2 ^a^ (-3.5~-3.0) |
| Both | 80+ | 2000-2015 | -2.3 ^a^ (-2.8~-1.8) |  | — | — |  | — | — |  | -2.3 ^a^ (-2.8~-1.8) |
| Male | 0-39 | 2000-2015 | -4.1 ^a^ (-5.1~-3.0) |  | — | — |  | — | — |  | -4.1 ^a^ (-5.1~-3.0) |
| Male | 40-49 | 2000-2015 | -4.9 ^a^ (-5.4~-4.4) |  | — | — |  | — | — |  | -4.9 ^a^ (-5.4~-4.4) |
| Male | 50-59 | 2000-2015 | -3.2 ^a^ (-3.9~-2.5) |  | — | — |  | — | — |  | -3.2 ^a^ (-3.9~-2.5) |
| Male | 60-69 | 2000-2003 | -7.1 ^a^ (-9.7~-4.6) |  | 2003-2010 | -0.6(-1.5~0.3) |  | 2010-2015 | -3.3 ^a^ (-4.5~-2.1) |  | -2.8 ^a^ (-3.5~-2.2) |
| Male | 70-79 | 2000-2004 | -4.9 ^a^ (-5.9~-3.8) |  | 2004-2015 | -2.8 ^a^ (-3.0~-2.5) |  | — | — |  | -3.3 ^a^ (-3.6~-3.0) |
| Male | 80+ | 2000-2015 | -3.2 ^a^ (-3.8~-2.6) |  | — | — |  | — | — |  | -3.2 ^a^ (-3.8~-2.6) |
| Female | 0-39 | 2000-2015 | -2.3 ^a^ (-3.1~-1.4) |  | — | — |  | — | — |  | -2.3 ^a^ (-3.1~-1.4) |
| Female | 40-49 | 2000-2015 | -4.3 ^a^ (-4.9~-3.7) |  | — | — |  | — | — |  | -4.3 ^a^ (-4.9~-3.7) |
| Female | 50-59 | 2000-2015 | -5.5 ^a^ (-6.2~-4.7) |  | — | — |  | — | — |  | -5.5 ^a^ (-6.2~-4.7) |
| Female | 60-69 | 2000-2004 | -6.8 ^a^ (-9.6~-3.9) |  | 2004-2010 | -1.8(-3.9~0.3) |  | 2010-2015 | -5.4 ^a^ (-7.4~-3.3) |  | -4.4 ^a^ (-5.5~-3.2) |
| Female | 70-79 | 2000-2015 | -3.5 ^a^ (-3.8~-3.1) |  | — | — |  | — | — |  | -3.5 ^a^ (-3.8~-3.1) |
| Female | 80+ | 2000-2015 | -1.8 ^a^ (-2.2~-1.4) |  | — | — |  | — | — |  | -1.8 ^a^ (-2.2~-1.4) |

Abbreviations: GEC, Gastric and esophageal cancer; APC, annual percent change; 95% CI, 95% confidence interval; AAPC, average annual percent change.

^a^ the average annual percent change is significantly different from zero (P<0.05).

**Table S2** JoinPoint analysis of GEC age-specific mortality trends by gender, 2000 to 2015.

| Sex | Age  (year) | Trend 1 | |  | Trend 2 | |  | Trend 3 | |  | 2000-2015 |
| --- | --- | --- | --- | --- | --- | --- | --- | --- | --- | --- | --- |
|  |  | Period | APC  (95%CI) (%) |  | Period | APC  (95%CI) (%) |  | Period | APC  (95%CI) (%) |  | AAPC  (95%CI) (%) |
| Both | 0-39 | 2000-2015 | -3.7 ^a^ (-4.8~-2.5) |  | — | — |  | — | — |  | -3.7 ^a^ (-4.8~-2.5) |
| Both | 40-49 | 2000-2003 | -9.3 ^a^ (-11.5~-7.1) |  | 2003-2013 | -4.6 ^a^ (-5.0~-4.2) |  | 2013-2015 | -12.2 ^a^ (-16.4~-7.9) |  | -6.6 ^a^ (-7.3~-5.9) |
| Both | 50-59 | 2000-2015 | -4.6 ^a^ (-5.0~-4.2) |  | — | — |  | — | — |  | -4.6 ^a^ (-5.0~-4.2) |
| Both | 60-69 | 2000-2003 | -9.0 ^a^ (-11.5~-6.6) |  | 2003-2011 | -2.4 ^a^ (-3.1~-1.7) |  | 2011-2015 | -4.7 ^a^ (-6.3~-3.1) |  | -4.4 ^a^ (-5.0~-3.7) |
| Both | 70-79 | 2000-2015 | -3.7 ^a^ (-4.1~-3.3) |  | — | — |  | — | — |  | -3.7 ^a^ (-4.1~-3.3) |
| Both | 80+ | 2000-2015 | -1.8 ^a^ (-2.5~-1.2) |  | — | — |  | — | — |  | -1.8 ^a^ (-2.5~-1.2) |
| Male | 0-39 | 2000-2013 | -3.2 ^a^ (-4.3~-2.0) |  | 2013-2015 | -21.1 ^a^ (-36.9~-1.3) |  | — | — |  | -5.8 ^a^ (-8.4~-3.1) |
| Male | 40-49 | 2000-2003 | -9.3 ^a^ (-13.0~-5.6) |  | 2003-2013 | -4.6 ^a^ (-5.3~-3.9) |  | 2013-2015 | -14.2 ^a^ (-20.9~-7.0) |  | -6.9 ^a^ (-8.0~-5.7) |
| Male | 50-59 | 2000-2015 | -3.9 ^a^ (-4.3~-3.4) |  | — | — |  | — | — |  | -3.9 ^a^ (-4.3~-3.4) |
| Male | 60-69 | 2000-2003 | -9.0 ^a^ (-11.2~-6.7) |  | 2003-2011 | -1.8 ^a^ (-2.5~-1.2) |  | 2011-2015 | -4.2 ^a^ (-5.7~-2.7) |  | -3.9 ^a^ (-4.5~-3.3) |
| Male | 70-79 | 2000-2003 | -7.1 ^a^ (-9.9~-4.2) |  | 2003-2015 | -2.9 ^a^ (-3.3~-2.6) |  | — | — |  | -3.8 ^a^ (-4.3~-3.2) |
| Male | 80+ | 2000-2015 | -2.5 ^a^ (-3.2~-1.8) |  | — | — |  | — | — |  | -2.5 ^a^ (-3.2~-1.8) |
| Female | 0-39 | 2000-2015 | -3.1 ^a^ (-4.6~-1.5) |  | — | — |  | — | — |  | -3.1 ^a^ (-4.6~-1.5) |
| Female | 40-49 | 2000-2015 | -5.4 ^a^ (-6.0~-4.7) |  | — | — |  | — | — |  | -5.4 ^a^ (-6.0~-4.7) |
| Female | 50-59 | 2000-2015 | -6.4 ^a^ (-7.1~-5.8) |  | — | — |  | — | — |  | -6.4 ^a^ (-7.1~-5.8) |
| Female | 60-69 | 2000-2015 | -5.1 ^a^ (-5.7~-4.6) |  | — | — |  | — | — |  | -5.1 ^a^ (-5.7~-4.6) |
| Female | 70-79 | 2000-2015 | -4.4 ^a^ (-4.8~-3.9) |  | — | — |  | — | — |  | -4.4 ^a^ (-4.8~-3.9) |
| Female | 80+ | 2000-2004 | -5.1 ^a^ (-9.3~-0.8) |  | 2004-2009 | 1.7(-2.8~6.4) |  | 2009-2015 | -3.9 ^a^ (-6.2~-1.5) |  | -2.4 ^a^ (-4.2~-0.6) |

Abbreviations: GEC, Gastric and esophageal cancer; APC, annual percent change; 95% CI, 95% confidence interval; AAPC, average annual percent change.

^a^ the average annual percent change is significantly different from zero (P<0.05).
